# Pre-clerkship medical students’ perceptions of medical professionalism

**DOI:** 10.1186/s12909-019-1629-4

**Published:** 2019-07-01

**Authors:** Danielle Reimer, Ross Russell, Bertha Ben Khallouq, Christine Kauffman, Caridad Hernandez, Juan Cendán, Analia Castiglioni

**Affiliations:** 1 Mayo Clinic Florida, Jacksonville, Florida, USA; 2 Baylor Scott and White Health, Temple, TX USA; 3 College of Medicine, University of Central Florida, 6850 lake Nona Blvd, Orlando, FL 32827 USA

**Keywords:** Professionalism, Medical education, Medical education-professionalism, Identity formation

## Abstract

**Background:**

Professionalism instruction and assessment is a core component of medical education, and essential for professional identity formation (PIF). Thus, understanding the socialization of medical students to the values of the profession (i.e., medical professionalism), and how these may evolve, warrants continued understanding.

**Methods:**

The purpose of this study was to examine and compare pre-clerkship (first and second year) medical students’ perceptions of professionalism. First and second year medical students participate in this study. This was a two-phase mixed-methods cohort study conducted across two academic years (2014–2015 and 2015–2016). In Phase I, first and second year medical students participated in a nominal group technique (NGT) session. NGT data was analyzed qualitatively to generate a card-sorting exercise of professionalism attributes for Phase II. In Phase II, data from the sorting task was analyzed using Principle Component Analysis (PCA).

**Results:**

The PCA for first year students derived a 7-factor solution. Factors (i.e., *professionalism domains*) identified were: *Self-management and patient-centeredness, ethics and professional reputation, dependability, self-awareness and self-improvement, image, proficiency and lifelong learning* and *integrity*. The PCA for second year students derived a 5-factor solution; factors identified were: *“Good Doctor” attributes, responsibility, ethics, innovation and self-improvement* and *unbiased.*

**Conclusions:**

Identification and organization of attributes into an overarching professionalism mental model provide a window into the active reconstruction of students’ professional identity during the nascent stages of medical education. M1 professionalism domains were more consistent with the conventional professional image of the physician (e.g. *Ethics and Professional reputation, Dependability, Integrity*), whereas, M2 domains reflected a more global view (e.g., *“Good Doctor” attributes*, *Responsibility*, *Ethics*). This study provides a lens into the dynamic nature of students’ PIF and encourages educators to evaluate PIF pedagogy at their own institutions.

## Background

Medical professionalism is a core clinical competency for medical students, trainees and practice physicians; thus, modalities and best practices for teaching have become an important component of medical education [1–4]. Over the past several decades, professionalism curricula have been described in the literature and have demonstrated the need and importance of deeper ethical and humanistic reflection, rather than relying on superficial observation and assessment of behaviors alone [5]. As a result, medical educators are tasked with designing curricula that teaches students the core elements of medical professionalism, while also effectively modeling what they teach. In 1995, the Association of American Medical Colleges (AAMC) published the *Assessment of Professionalism Project,* providing a resource with specific examples of behaviors that define medical professionalism for students, trainees and the practicing physician [1]. These behavior sets have served as a guide for medical educators tasked with measuring and assessing professionalism in medical education. However, medical schools differ in their strategies of instruction and evaluation [1–4].

Recently, there has been an increasing emphasis on the process of professional identity formation (PIF) and the need to better understand students’ implicit and explicit understanding of medical professionalism [6]. Identity formation is defined as the changing professional concept self-based on the integration of knowledge, skills, beliefs, values and experiences [7]. Holden et al., applied the concept of identity formation in medicine as the developmental and complex process of the transformation of a lay person into a physician as one begins to establish their unique core values, morals, ethical principles and self-awareness [8, 9]. PIF in medical education has been shown to be most transformative during the transition from undergraduate education to medical education, clinical years, experience with the business of medicine and finally exposure to the practice of physicians [10].

The University of Central Florida College of Medicine (UCFCOM) has an integrated longitudinal curriculum that incorporates medical professionalism instruction and assessment throughout the four-year M.D. program. The UCFCOM MD Curriculum Committee charged a task force to define an evidence-based professionalism framework that incorporates the AAMC behavior sets and the work of many others [2, 11–22]. The UCFCOM professionalism framework is composed of twenty-five elements that map into six domains [23]. During the first two years, professionalism is taught explicitly. Students participate in a course series, the Making of a Physician (MOP) Program, during which they have opportunities to work in small groups with faculty mentors and discuss topics that nurture professional growth, such as humanism, empathy, and cultural competence.

MOP begins with a session dedicated to medical professionalism and includes relevant pre-session readings, such as Swick’s Towards a Normative Definition of Medical Professionalism [24]. During the small group session students discuss how the professional responsibilities of physicians apply to medical students. Students are also presented with scenarios of common professionalism lapses by medical students and discuss contributing factors as well as potential strategies to avoid these situations. The principles of medical professionalism are revisited throughout subsequent MOP sessions. Another integral component of the pre-clerkship curriculum is the Community of Practice (COP) Program, a longitudinal preceptorship experience, where students work side-by-side practicing physicians providing an authentic clinical context to promote deeper learning, professional identity formation, and adoption of the values of the profession.

Pre-clerkship formative and summative assessments include multiple-choice items on written exams related to knowledge of the foundational principles of medical professionalism and medical ethics, and cover topics such as patient confidentiality, maintaining appropriate relationships with patients, access to care, just distribution of finite resources, and professional responsibility. Students’ professionalism in COP sessions is assessed by their clinical preceptors using a professionalism rubric. Additionally, clinical skills encounters with standardized patients allow for professionalism assessment through direct observation of the student’s behaviors; here, students are provided feedback from the patient’s perspective, as well as from faculty and staff. Moreover, faculty emphasize with students how observed behaviors during the pre-clerkship years (interaction with patients, faculty, staff and fellow student) can be a surrogate for later behavior in patient care.

Professionalism instruction in the latter two clinical clerkship years of the MD program relies on mentoring and role modeling, where students have an opportunity to model the behaviors they observe from the practicing physicians they work with. Medical Professionalism is expected of all clinical faculty, and emphasized in UCFCOM’s faculty development program. Discussions with fellow students and clerkship directors give students an opportunity to debrief observed behaviors of clinical preceptors; egregious behaviors are reported to the Dean for Students. In addition, at the end of each rotation students complete an anonymous preceptor evaluation. Similarly, clerkship faculty assess students’ professionalism through a clerkship evaluation rubric.

In this study, we investigate how UCFCOM formal, informal and hidden curricular experiences impact student’s professional identity formation.

## Methods

### Study design and participants

This was a two-phase mixed-methods cohort study conducted across two academic years (2014–2015 and 2015–2016) at the University of Central Florida College of Medicine (UCFCOM). Phase I of this study was qualitative (focus group; specifically, nominal group technique) and Phase II was quantitative (sorting exercise). This was a convenient sample of matriculated first (*n* = 120) - and second (*n* = 120) -year students (M1and M2, respectively). All students (*N* = 240) were invited to participate via institutional email during the week of orientation. The first ten M1 and M2 students (*N* = 20) to respond via email were invited to participate in Phase I. Phase I was repeated the subsequent academic year recruiting an additional ten M1 and ten M2 students to participate. Phase II took place in the second year of the study, where all matriculated M1 and M2 students were invited to participate in an online, anonymous, card-sorting exercise via email. Participation was voluntary and demographic characteristics of participants were not collected. Participants were compensated $10 for the NGT session and $5 for the card sorting activity (i.e., 20 participants could receive up to $15). All participants received written informed consent information. Furthermore, the Institutional review board at the UCF reviewed and approved this study (SBE-14-10,403).

#### Phase I: nominal group technique (NGT)

NGT is a structured interview technique used to generate ideas about a topic from a group of individuals (in our case perceptions of medical professionalism). NGT generates a high number of quality ideas from stakeholders, while also allowing individuals to have equal input and the ability to express and prioritize their ideas. NGT requires four steps: 1) participants silently generate responses to a question, 2) in a robin-round fashion, responses are recorded and shared 3) recorded responses are discussed for clarification, and 4) participants vote on the importance of responses [25, 26].

#### NGT procedure

Four NGT sessions with M1 and M2 students were conducted (two per class) during the first week of the academic years. The same moderator conducted all four NGT sessions. At the beginning of the sessions, the moderator gave the students a brief overview of the NGT process and informed them that the purpose of the session was to learn and share their perceptions of medical professionalism.

Students worked silently for 8 min to generate a list of clear and concise responses to the following prompt: *“What is medical professionalism? Think broadly about factors which define medical professionalism and also factors which affect physicians’ (and physicians in training) professional behavior”* (NGT step 1). For the next 15 min, students presented one of their responses, without providing any rationale. To ensure equal contribution, students shared in a round-robin fashion (NGT step 2). Once all responses were shared and recorded, students identified repetitive or compound ideas: Students discussed group responses and voted to combine and separate responses. (NGT step 3). As a final step, students independently and anonymously ranked the group’s responses in order of personal importance by assigning a weighted ballot (ranging from 1 to 5) to the top five responses that “*contribute the most to medical professionalism*” (NGT step 4). The final product of each session was a prioritized, ranked list of professionalism attributes.

#### NGT: data analysis

Student-derived professional attributes from the NGT sessions were combined across academic years, separately for M1 and M2 students. Three of the investigators (AC, CK, and CH) used recursive abstraction to summarize the number of professional attributes. That is, similar statements were combined and repetitive statements eliminated. At the end of this exercise, the investigators had two shorter lists of *professionalism attributes*: For M1 students (51 attributes) and for M2 students (66 attributes), see Tables 1 and 2. These *professionalism attributes* were used in Phase II of this project.

**Table 1:** M1- Medical Professionalism Attributes

Respectful	Respecting confidentiality
Culturally competent	Maintain appropriate relationships with patients
Unbiased	Not being under the influence of drugs or alcohol
Works well on a team	Inspiring confidence in your patient, peers, and students
Efficiency	Not being judgmental
Good networking skills	Maintain a neat and tidy appearance
Organized work space	Helping to eliminate health disparities
Leadership qualities	Separation of work and personal life
Mature	Skillful and proficient in their specialty
Appreciation of others time	Seeking self-improvement and evaluations from others to identify deficits
Integrity	Reporting instances where rules of standards are not upheld
Dependable	Maintaining a patient’s autonomy
Having humility	Attentive to patient needs
Consistency	Working towards improving the profession as a whole
Even tempered remaining calm under stress	Appropriate use of social media and social interaction
Putting patient care first	Being courteous
Responsible use of knowledge or influence	Mindfulness of the way the public perceives medicine
Using proper communication when interacting with patient	Informs patients before conducting any tests or examinations
Practicing using the most current information	Honest but not callous
Honest and forthcoming when speaking to patients	Spending more time than required if necessary
Can clearly communicate complex ideas	Displaying appropriate body language
Empathetic to patient situation	Wash hands
Listens and shows interest in patients	Willing to give and receive feedback
Able to navigate through conflict without escalating it	Knowing what is appropriate in different situations
Supporting of other clinicians and their decisions	
Respectful toward death and disease	
Ethical standards, behavior and habits that promote safe treatment of patients

**Table 2:** M2- Medical Professionalism Attributes

Dependable	Non-maleficence
Responsible	Responsiveness
Respecting colleagues and their opinions	Helping colleagues and peers when they need it
Treating all patients equally despite gender, race or religion	Able to adjust well to different situations
Putting the patient first	Intrinsically motivated
Competent	Time management
Knowledgeable	Keeping personal beliefs separate from medical advice
Acting with Integrity	Tolerance
Empathy	Able to work well with a spectrum of people
Commitment to providing quality care	Confident but also modest
Dedicated to the work	Acknowledging your own limitations
Team player; knowing when to lead and when to support	Timeliness
Beneficence	Holding yourself to a high standard at all times. Maintaining a professional …
Compassionate	How you present yourself (attire, language used, body language)
Attentive listener	Admitting mistakes. Accepting responsibility for success and failures
Avoiding conflicts of interest	Inspire confidence in others including patients and families (giving people a …
Culturally competent	Giving people your full attention and not being distracted
Discipline	Having the courage to stand up for yourself and others when you feel there …
Open-mindedness	Being non-judgmental and objective
Maintaining trust and confidentiality	Spread your wealth of knowledge
Appropriate interactions with other members of the patient care team	Innovative - Always seeking to advance or improve medical practice/patient care
Considerate of patients’ rights and needs	Setting appropriate boundaries for patients/physicians
Preventing external factors from interfering with patient interactions	Respecting the authority of your superiors and the boundaries set by them
Caring about the education of patients	Attitude of consideration for ethics and ethical principles
Communicates well	Holding others accountable to maintain professionalism
Having a good attitude	Showing consideration and eliminating inequality for marginalized groups
Managing stress	Strive to continue and maintain your medical education and knowledge
Maintaining moral code	Asking for feedback from the people around you and learning from it
Speaking respectfully	Never showing up for work impaired by drugs or alcohol
Maintaining composure when dealing with all patients	Being a positive role model for your patients in their moments of weakness
Looking over things; double-checking	Showing dignity to your patients in their moments if weakness
Being humble	Recognizing that taking care of a patient’s health is a privilege and act … .
Patience	
Safety in performing medical procedures	

#### Phase II: card-sorting exercise

A card sorting exercise was used to investigate the complexities of student’s professional identity formation. Card sorting is a well-accepted, participant-centered, consumer oriented technique, in which study participants group individual statements according to criteria that make sense to them. This method uncovers how the target audience’s values and perspectives are structured, and it serves to create an information architecture. Card sorting reduces bias and improves the generalizability of results [27]. In our study, the goal of the participants was to sort *professionalism attributes*.

#### Card sorting exercise procedure

M1and M2 students who volunteered to participate in Phase II (42 and 58, respectively), completed a confidential electronic card-sorting exercise using Qualtrics software (Qualtrics, Provo, UT). M1 and M2 students saw separate sets of cards (*professionalism attributes;* Tables 1 and 2) and the instructions were as follows: “*On the left you will find a stack of professionalism attributes generated by first year [second year] medical students at UCF COM. We’d like you to sort them into groups that make sense to you. There is no right or wrong answer, and no particular order or ranking. Just do what comes naturally and group the attributes as you see them fit together*. *Sort into two to ten piles; with each pile containing at least two cards*.”

#### Data analysis

Principal component analysis (PCA) is a multivariate technique used to identify patterns or groups in a data matrix of high dimension, the mathematical expressions in PCA extract important information and express the results as factors based on PCA derived loading values. Two PCAs were conducted, separately for M1 and M2 student professional attributes. Due to the exploratory nature of these models, rotations were not applied. Consistent with PCA standards, factor loadings with a value of .55 or higher were included [28]. Factors with an Eigenvalue > 1 were used. The Statistical Package for the Social Sciences (SPSS v.23.0: IBM; Armonk, NY) was used for data analysis. Investigators (AC, CK, CH and DR) examined the factor solutions for each class, discussed possible themes and reached a consensus on a label for each factor. To enhance readability by educators and non-statisticians, hereafter, factors solutions are referred to as professionalism domains.

## Results

**Fig. 1:**
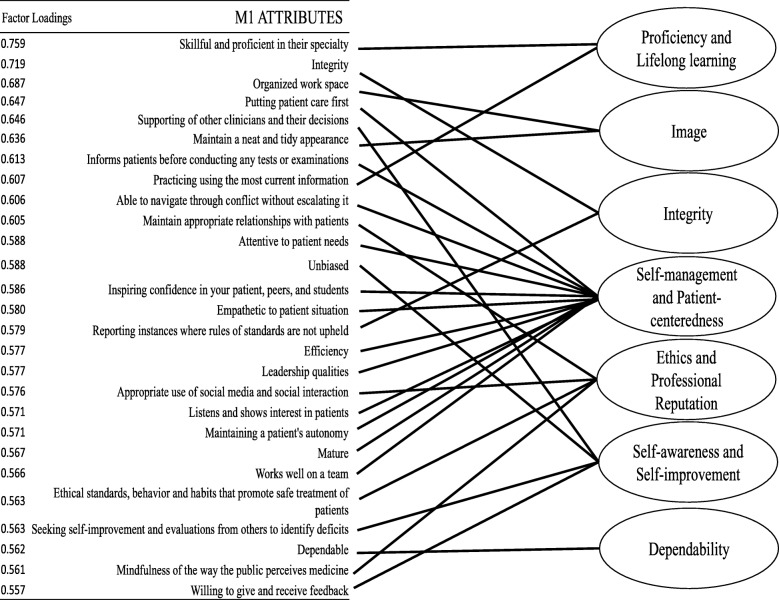
Visual representation of 27 attributes identified by M1 students with individual factor loadings and placement in the 7 domains

Forty-two (42) M1 and fifty-eight (58) M2 students completed the electronic card-sorting exercise, representing 35 and 48% response rate respectively. M1 PCA derived seven professionalism domains composed of 27 professionalism attributes with factor loadings ranging from (.56 to .76*).* Professionalism domains identified were: *Self-management and patient-centeredness* (12-items), *Ethics and professional reputation* (4-items), *Dependability* (1-item), *Self-awareness and self-improvement* (4-items), *Image* (2-items), *Proficiency and lifelong learning* (2-items) and *Integrity* (2-items), see Fig. 1.

**Fig. 2:**
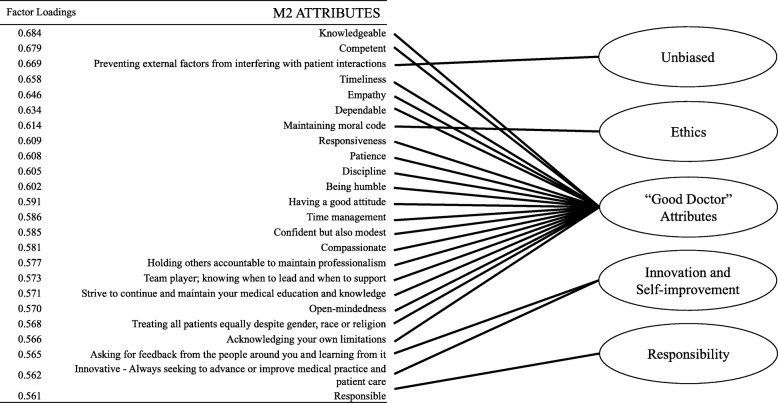
Visual representation of 24 attributes identified by M2 students with individual factor loadings and placement in the 5 domains

The M2 students PCA derived five professionalism domains comprised of 24 professionalism attributes with factor loading ranging from (.56 to .68). *Professionalism domains* were: *“Good Doctor” attributes* (18-items), *Responsibility* (1-item), *Ethics* (1-item), *Innovation* and *Self-improvement* (2-items) and *Unbiased* (1-item), see Fig. 2.

As a final step, investigators examined the professionalism domains identified by each class and mapped these to the AAMC’s defined professionalism behavior sets. For alignment of M1 and M2 domains with the AAMC behavior sets see Table 3.

**Table 3:** Comparison of Student-Identified Domains with AAMC Professional Behavior Sets

AAMC Behavior Sets	M1	M2
Physicians subordinate their own interests to the interests of others	x	
Physicians adhere to high ethical and moral standards	x	x
Physicians respond to societal needs, and their behaviors reflect a social contract with the communities served	x	x
Core humanistic values, including honesty and integrity, caring and compassion, altruism and empathy, respect for all	x	x
Physicians exercise accountability for themselves and for their colleagues	x	x
Physicians recognize when there is a conflict of interest to themselves, their patients, their practice	x	x
Physicians demonstrate a continuing commitment to excellence	x	
Physicians exhibit a commitment to scholarship and to advancing their field		x
Physicians are able to deal effectively with high levels of complexity and uncertainty		
Physicians reflect critically upon their actions and decisions and strive for improvement in all aspects of their work	x	x
Professionalism incorporates the concept of one’s moral development		
Professionalism includes one’s responsibility to the profession as a healer		
Professionalism includes receiving and responding to critiques from peers, students, colleagues, and peers	x	x
Physicians must demonstrate sensitivity to multiple cultures	x	x
Physicians must maintain competence in the body of knowledge for which they are responsible for. Commitment to lifelong learning	x	x
Altruism and dutifulness	x	x

Alignment of medical professionalism domains identified by first and second year medical students at UCF College of Medicine with the AAMC Professionalism Behavior Sets

## Discussion

In recent years, there has been a heavy focus on the importance of professionalism in medical education and developing effective professionalism curricula. Previous studies have shown that medical students’ understanding of professionalism improves and evolves over time through self-reflection, experience and exploration [3, 29]. This study differs from previous literature in that it focuses on the dynamic process of professional identity formation during the early pre-clerkship years. The professionalism domains identified here contribute to the understanding of medical student’s professionalism mental model at the beginning stages of their education.

First year students, with minimal to no exposure to professionalism curriculum identified seven domains: *Self-management and patient-centeredness, Ethics and professional reputation, Dependability, Self-awareness and self-improvement, Image, Proficiency and lifelong learning,* and *Integrity*. On the other hand, second year students, with one year of curriculum exposure identified 5 domains: “*Good Doctor” Attributes, Responsibility, Ethics, Innovation and self-improvement,* and *Unbiased*. Results suggest that students drawn to medical education recognize fundamental core values of the medical profession even at the earliest level of their medical education. It appears that students arrive to medical school with a preconceived notion of the professional expectations of a physician.

M1 and M2 students both identified domains focused on the intrinsic qualities of physicians (*Self-management and Patient-centeredness* and *“Good Doctor” attributes*, respectively). A closer look at the attributes within the domains, suggests that M1 students focused on the intrinsic qualities as a means of self-management in both a personal and professional role of serving the patient. This emphasis on patient-centered care is not present in the professionalism mental model constructed by M2 students. This difference may be attributed to many factors, for instance, students are beginning to experiment with a provisional self as they move forward in developing their own professional self-concept [9, 10]. It is also important to consider the culture of the academic institution, as well as the impact of course curricula. During the pre-clinical years, students are heavily focused on their studies, working to master their demanding coursework and completing required clinical experiences. This stressful environment of the preclinical years may explain the manifestation of a student-centered culture.

As stated previously, both the M1 and M2 professionalism mental models identified domains focusing on the intrinsic qualities of a physician; however, the M2 students were better able to articulate the role of a physician within a singular domain. These intrinsic professionalism attributes were consolidated into one domain representative of the personal qualities of the ideal “good doctor.” It is unclear if this change between M1 and M2 students signifies a true change of students’ professionalism perceptions or if this simply reflects the improved ability of students to categorize professional attributes after exposure to our curriculum.

Another core professionalism principle of ethics was identified by both M1 and M2 classes (*Ethics and Professional Reputation* and *Ethics,* respectively); however, first year students emphasized a relationship between ethical standards and the importance of the professional reputation of a physician. First year students who identified *Image* as a domain highlight the idea of professional reputation once again. This domain focused on the professional reputation of a physician from a superficial point of view, valuing organization and appearance. This idea may align with a societal view of medical professionalism, with the “clean white coat” serving as the pre-eminent symbol of the physician.

Mapping of the student-identified domains with the AAMC defined professionalism behavior sets suggests more similarities than discrepancies between first- and second-year students’ mental models, with most the behavior sets identified by both classes. As stated previously, a noticeable difference is the loss of focus on patient-centered care for second year students. Both classes emphasize the important of physician competency and self-improvement; however, second year students also emphasize the important role of advancement of the field. Notably, M1 and M2 students failed to identify domains aligned with the following AAMC behavior sets: physicians able to deal effectively with high levels of complexity and uncertainty, moral development or responsibility to the profession as a healer. It is plausible that students will recognize these behaviors after more clinical exposure and preceptor role-modeling during the clerkship years [11]. Faculty development will be critical to align the importance of professionalism education and assessment across the educational continuum.

Some of the differences between the way M1 and M2 students viewed professionalism were small, while others were more notably different. However, evaluating and exploring these differences gives us insight into the changing professional identity of these medical students as they progress through the preclinical years of medical education. Professional identity development does not always occur in linear fashion and larger longitudinal studies are an important next step to fully understand the multiple, complex and embedded processes of PIF as medical students’ progress through medical school training [29, 30].

Previous studies have attempted to understand medical students’ PIF. Lown at al. used a similar consumer-based approach to explore M1 students’ perceptions of their personal and professional development as they entered medical school in the United Kingdom [31]. Our results contribute to the existing literature by providing a current view of millennial medical students’ PIF, focusing on pre-clerkship students, and attempting to further understand how this identity evolves in the first two years of education.

Some limitations of this study should be noted. It was conducted at a single institution and included a limited number of participants from each class; thus, results may not be generalizable to other schools. Furthermore, the convenient sample introduces sampling bias and limits external validity. Given the exploratory nature of this research and the guidelines for using NGT, our four NGT groups likely generated a reasonable list of responses for our institution. Multi-institutional expansion of this study would increase the external validity of our findings. Our study focused on pre-clerkship students, we anticipate expansion to include third and fourth-year medical students to better understand the longitudinal changes and impact of the current medical training on students’ professional identity formation at a more advanced stage in the medical education continuum. PCA serves as a preliminary analysis and it would be prudent to apply rotations to this data in future studies to further analyze the structure and identify other data patterns. Nonetheless, our findings support other studies that have shown that medical students’ understanding of professionalism improves and evolves over time through self-reflection, experience and exploration.

## Conclusion

Continuous efforts to investigate the transformative process and the evolving professionalism perceptions of students across academic institutions are needed. Expanding the knowledge of PIF in the medical education community to evaluate current PIF pedagogy, including current medical training, the culture of the institution and any hidden curricula, will help further the understanding of the critical process that transforms the lay person into a physician [32, 33].

## Data Availability

The datasets used and/or analyzed during the current study are housed in UCF College of Medicine server and available from the corresponding author on reasonable request.

## Abbreviations

AAMC: American association of medical colleges
COP: Community of practice
FIRE: Focused inquiry research experience
M1: First year medical student
M2: Second year medical student
MOP: Making of the physician
NGT: Nominal group technique
PCA: Principal component analysis
PIF: Professional identity formation
SPSS: Statistical package for social sciences
UCF: University of Central Florida
UCFCOM: University of Central Florida College of Medicine
